# Horizontal Transfer of a Novel Soil Agarase Gene from Marine Bacteria to Soil Bacteria via Human Microbiota

**DOI:** 10.1038/srep34103

**Published:** 2016-10-19

**Authors:** Tao Song, Hui Xu, Congchong Wei, Tengfei Jiang, Shishang Qin, Weijia Zhang, Yu Cao, Chao Hu, Fan Zhang, Dairong Qiao, Yi Cao

**Affiliations:** 1 Microbiology and Metabolic Engineering of Key Laboratory of Sichuan Province, College of Life Sciences, Sichuan University, Chengdu, 610065, P. R. China; 2 National Engineering Research Center for Biomaterials, Sichuan University, Chengdu, 610065, P. R. China

## Abstract

Seaweed is receiving an increasing amount of attention as a “sea vegetable”. The microbiota of coastal populations may acquire seaweed associated enzymes through marine food. Several agarases have been found in non-marine environments; however, their origin is unknown. In this study, a hypothetical protein, Aga1, was identified as an agarase from an inland soil agar-degrading bacterium, *Paenibacillus* sp. SSG-1.Having low similarity to known glycoside hydrolases, Aga1 may be a distant member of the glycoside hydrolase family 86. Aga1 has good pH stability (pH 3–11) and is stable in the presence of various metal ions. Aga1 is an exo-type β-agarase that produces NA 4 (neoagarotetraose) and NA 6 (neoagarohexaose) as its main products. In addition, Aga1 may be a cell-surface-binding protein. The bioinformatic analysis showed *aga1* may have been transfered together with its surrounding genes, from marine bacteria to soil bacteria via human microbiota. The use of seaweed as food and the disposal of human faeces or saliva were the most likely reasons for this gene transfer pathway. Notably, the results also indicated that microbes from inland humans may degrade agar and that these microbes may have acquired seaweed associated genes because of increased seaweed in diets.

In 2012, the world’s annual production of seaweed reached 23.8 million tons, which is 3.5 times more than that produced in the 1990s^1^. In recent decades, seaweed has become increasingly popular as food, not only in Asian countries, but also in western countries^2^,^3^,^4^,^5^. Compared to traditional crops, seaweed has many superior characteristics, such as being fertilizer-free and irrigation-free and having no land conflicts with traditional agricultural crops^6^,^7^. Considering its nutrient composition, including amino acids and fatty acids, seaweed is a promising food source.

Red seaweed, as an important marine plant, is widely used as food. Agar is the main component of red seaweed, and it consists of 3-O-linked β-D-galactopyranose and 4-O-linked α-3,6-anhydro-L-galactopyranose linked to sulfate groups, methyl groups or pyruvic acid acetal^8^. Agarase is the enzyme that degrades agar into oligosaccharides or monosaccharides^8^,^9^. Based on cleavage patterns, agarases may be classified as α-agarases, β-agarases and β-porphyranases^10^. According to the CAZy (carbohydrate-active enzymes) database, approximately only 40 agarases have been characterized^11^. Some agarases belong to existing glycoside hydrolase (GH) families, such as GH16^12^,^13^ and GH50^14^,^15^,^16^. Others have defined new families, such as GH86^17^,^18^, GH96^19^,^20^, GH117^21^,^22^ and GH118^23^,^24^. Compared with other well-studied glycoside hydrolases, such as cellulases or xylanases^25^, the number of agarases is small. Identifying and studying new agarases is essential.

Until now, most reported agarases have come from marine environments^13^,^14^,^15^,^16^,^17^,^19^,^23^. Agar-degrading bacteria have also been found in different non-marine environments, such as soil^26^,^27^,^28^, plant endogenous environments^29^, rivers^30^ and even the human gut^31^,^32^. It is interesting that agarase exists in environments containing nearly no seaweed. It has been reported that coastal human microbiota may acquire glycoside hydrolases from the marine environment through food connections^33^. It is not known where non-marine source agarases come from, although marine environments or soil environments are possibilities. An outstanding question is how non-marine agarases came into existence.

In our previous work, we purified and characterized a natural agarase from the agar-degrading soil bacteria *Paenibacillus* sp. SSG-1^27^. This natural agarase is a hypothetical protein in the genome of *Paenibacillus* sp. SSG-1. Furthermore, this hypothetical protein (Aga1) was synthesized, purified and characterized as agarase. Aga1 had low similarity to known agarases and was a distant member of GH86. A detailed biochemical characterization was conducted to determine the specific properties of Aga1. Bioinformatic analysis revealed that *aga1* may be the result of horizontal gene transfer from a marine environment to a soil environment via human microbiota.

## Results

### Cloning and identification of agarase *aga1*

Using the matched peptide sequences of SSG1a, a BLAST search against the *Paenibacillus* sp. SSG-1 genome identified one hypothetical protein, which was subsequently designated Aga1.

**Figure 1:**
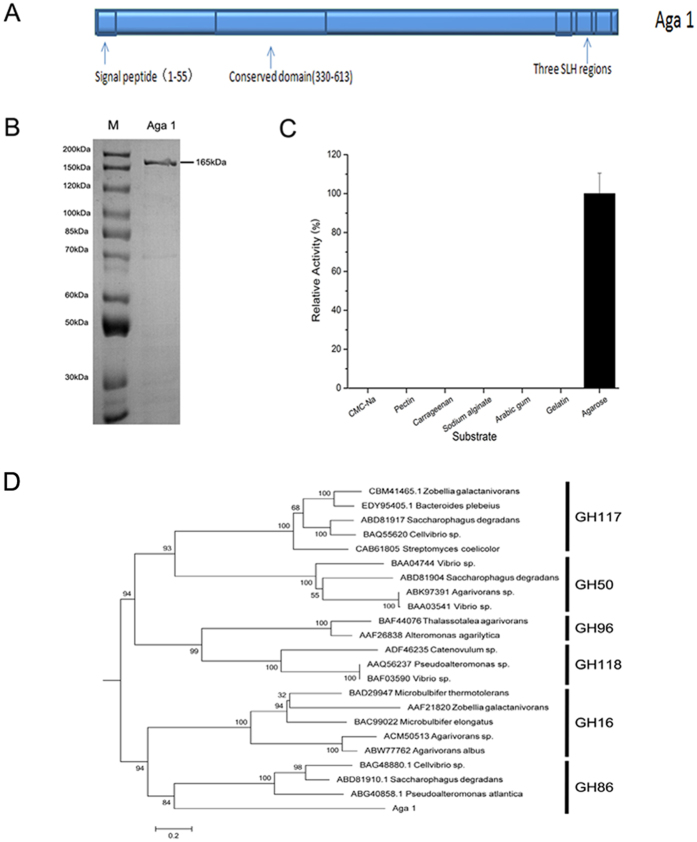
(**A**) Structure of Aga1 from Paenibacillus sp. SSG-1. SLH regions represent the S-layer homology regions. (**B**) SDS-PAGE and Coomassie staining of purified Aga1. (**C**) Aga1’s enzymatic activity against different substrates. (**D**) Phylogenetic tree between *aga1* and the characterized agarases. Numbers at nodes are levels of bootstrap support (%).

As demonstrated in Fig. 1A, Aga1 contains one signal peptide (1–55), a conserved region (330–613), and three S-layer homology (SLH) domains (1495–1536, 1554–1597 and 1624–1668).The signal peptide and SLH domains indicate that Aga1 is probably a secreted protein and may be located on the cell wall surface, which was supported by the subcellular location prediction results.

The recombinant protein Aga1, excluding the signal peptide and SLH domains, was inserted into the pET28a vector (His-tag fusion) and expressed in *E. coli* (DE3) as a soluble protein. SDS-PAGE showed the Aga1 protein had an apparent molecular mass of 165 kDa (Fig. 1B), which matched the calculated molecular mass of 166.3 kDa. Tandem mass analysis of the purified Aga1 confirmed that it was correctly expressed and purified (Supplemental Table S1).

As shown in Fig. 1C, Aga1 is an agarase that is active only in the presence of agarose. Aga1 had very low similarity (lower than 30%) to other glycoside hydrolases. The phylogenetic tree consisting of Aga1 and known agarases showed that Aga1 may be a distant member of GH86 (Fig. 1D).

### Biochemical analysis of agarase

**Figure 2:**
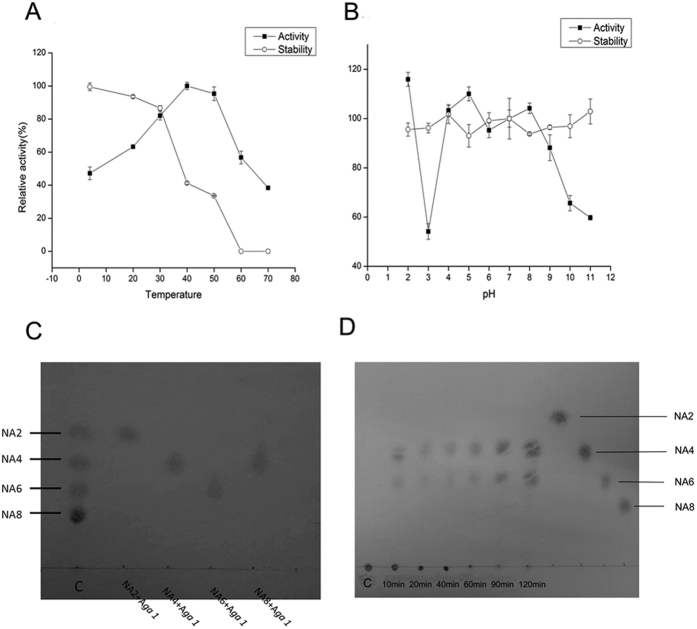
(**A**) Effects of temperature on stability and activity of Aga1. (**B**) Effects of pH on stability and activity of Aga1. Data are mean ± SD of three independent experiments. (**C**) TLC analysis of the catalytic property against the oligosaccharide. (**D**) The TLC analysis of the end products at different time. *NA2, NA4, NA6, NA8* represent the neoagarooctaose, neoagarotetraose, neoagarohexaose and neoagarooctaose, respectively.

Aga1 maintained over 40% of its activity across a wide range of temperatures (0–70 °C), and 50 °C was the optimal temperature for Aga1 (Fig. 2A). In addition, as shown in Fig. 2B, Aga1 showed strong stability over a wide pH range (pH 1–12). The decrease in Aga1 activity at pH 3.0 may be related to the pI of Aga1. Because the predicted pI of Aga1 was 4.5, it is possible that the real pI was close to pH 3.0, which caused this decrease. Meanwhile, most metal ions (1 mM) did not affect the activity of Aga1 (Supplemental Table S2). However, Cu^2+^ strongly inhibited its activity (37% activity).

As shown in Fig. 2C, Aga1 could not hydrolyse neoagarobiose (NA 2), neoagarotetraose (NA 4) and neoagarohexaose (NA 6). Neoagarooctaose (NA 8) was the smallest oligosaccharide that Aga1 could hydrolyse.

**Figure 3:**
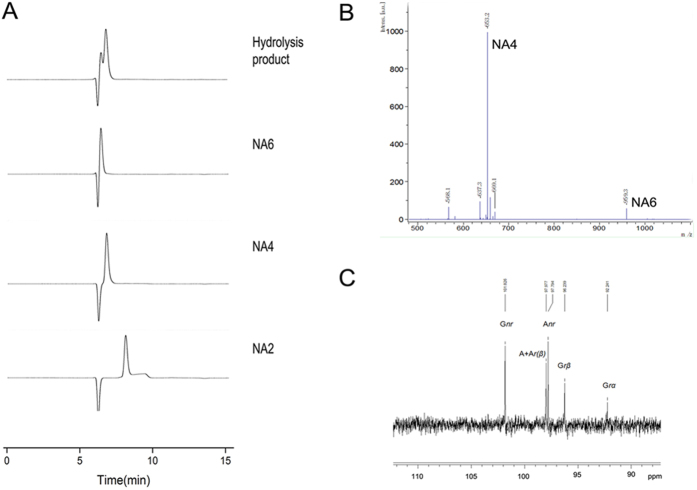
(**A**) The HPLC result of the hydrolysis product produced by Aga1. (**B**) MALDI-TOF mass result of the end products. (**C**) The ^13^C NMR of the end products. *G* β-D-galactopyranose, *A* 3,6-anhydro-a-L-aglactopyranose, *r* reducing end, *nr* non-reducing end, α α anomer, β β anomer.

TLC analysis of the end product showed that Aga1 hydrolysed agarose into two main products, which should be NA 4 and NA 6 according to the standards (Fig. 2D). These two products were also subjected to HPLC, and the results showed that they had the same retention times as NA 4 and NA 6 (Fig. 3A). Moreover, as shown in Fig. 3B, the MALDI-TOF mass spectrometry results of the end products showed two main peaks, i.e., 653.2 m/z and 959 m/z, which corresponded to [M + Na]+. These two peaks were attributed to NA 4 and NA 6, respectively. Taking these three results into consideration, the main hydrolysis products were NA 4 and NA 6.

To analyse the cleavage pattern of Aga1, ^13^C NMR was conducted. As shown in Fig. 3C, resonances at approximately 97.04 ppm and 93.05 ppm corresponded to the β and α anomeric carbons, respectively, of the galactose residues. Resonance at 90.72 ppm, the typical signal of an α-agarase, was not observed. Thus Aga1 is a β-agarase.

TLC (Fig. 2D) and HPLC analysis (Supplemental Fig. S1) showed that the amounts of NA 4 and NA 6 increased as the hydrolysis time increased, and no other oligosaccharides were observed during hydrolysis. Endo-agarase decomposed agarose in a random way and produced oligosaccharides with different degrees of polymerization during hydrolysis. As only two products were observed in the hydrolysis procedure, Aga1 was determined to be an exo-type agarase.

### Inland aga1 may be from inland human symbionts

To investigate the distribution of *aga1*, we searched for homologues of *aga1* in NCBI none-redundant database. Interestingly, almost all of *aga1*’s similar genes came from other class or phylum. Among the 208 genomes of *Paenibacillus* sp., *aga1* like gene only appeared in *Paenibacillus* sp. D14. This rare distribution of *aga1*’s similar gene in its own genus indicated that *aga1* may be a foreign gene. Meanwhile, the GC content, GC3s content, and Codon Bias Index of *aga1* were all significantly different from those of the genome (Table 1 and Supplemental Table S3). Additionally, the Relative Synonymous Codon Usage (RSCU) value of *aga1* was clearly distinct from the genome’s value (Supplemental Table S4). Thus, these evidences strongly indicated that *aga1* had been transfered horizontally from other microbes.

**Figure 4:**
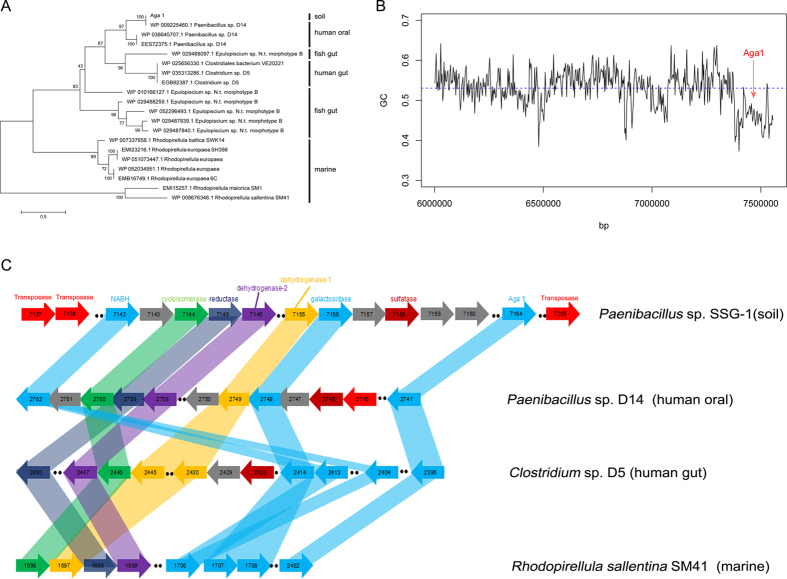
(**A**) Maximum likelihood tree of Aga1 and similar proteins. Numbers at nodes are levels of bootstrap support calculated from 100 bootstrap replicates (%). (**B**) The GC content change around the *aga1*. The red arrow indicates the position of *aga1*. The blue line represents the GC content of the whole genome of *Paenibacillus* sp. SSG-1. (**C**) Schematics of clusters containing *aga1* like genes in different species. Sequence related genes (higher than 30% identity) are linked. NABH: α-neoagarobiose hydrolase; *cycloisomerase*: 3,6-anhydro-L-galactonate cycloisomerase; *reductase*: 2,5-diketo-3-deoxy-L-galactonate 5-reductase; *dehydrogenase-1*: 3,6-anhydro-L-galactose dehydrogenase; *dehydrogenase-2*: 2-keto-3-deoxy-L-galactonate 5-dehydrogenase.

Given that *aga1* was from an inland soil environment that was far from the sea and contained almost no seaweed, it was difficult for the enzymes to evolve into agarases without a substrate, and the distant geographic position of the enzyme made gene transfer nearly impossible. As showed in Fig. 4A, *aga1* had a closer relationship with genes from symbiotic environments, other than those from marine environments. Meanwhile, we noticed that four *aga1* like genes came from inland people’s microbiota; i.e., from the human gut or human mouth. Human faeces are usually used as fertilizer, and discarding saliva is also common. Given that *aga1* was also from an inland location, it is reasonable to infer that *aga1* may come from inland human microbiota.

### Horizontal gene transfer linkage

Analysis of *Paenibacillus* sp. SSG-1’s genome showed that *aga1* was surrounded by other genes coding for agar, including α-neoagarobiose hydrolase (NABH), galactosidase. 3,6-anhydro-L-galactose (L-AnG) metabolic enzymes and sulfatase. All these genes were located in a region which had an atypical GC content value with the genome (Fig. 4B). Moreover, NABH, and galactosidase were also uncommon in *Paenibacillus* sp. Their closest homologues (>70% identify) were found in other microbes, such as *Clostridium* sp. D5, *Paenibacillus* sp. D14. As showed in Fig. 4A, *aga1* had a closer relationship with genes from human symbiotic environments. Thus, *aga1* and its surrounding genes in *Paenibacillus* sp. SSG-1 may have been horizontally transfered from other microbes, such as human oral or gut symbionts. As discussed above, the most possible mode for this transfer was the disposal of human faeces or saliva.

When the distribution of *aga1* like gene, NABH and galactosidase genes from human symbionts, *Paenibacillus* sp. D14 and *Clostridium* sp. D5, were investigated, we also found that these genes were rare in their corresponding genus. Meanwhile, the homologue of these genes could be found in the marine bacterium *Rhodopirellula sallentina* SM41. Conserved gene pair was also the indicator of horizontal gene transfer^34^. As showed in Fig. 4C, conserved gene pairs could be observed among the clusters. Gene pair, encoding for dehydrogenase-2 and reductase, was conserved between *Paenibacillus* sp. D14 (genes 2759 and 2758) and *Rhodopirellula sallentina* SM41 (genes 1698 and 1699). Gene pair, encoding for cycloisomerase and dehydrogenase-2, was conserved between *Clostridium* sp. D5 (genes 2446 and gene 2447) and *Rhodopirellula sallentina* SM41 (genes 1696 and 1697). Moreover, transposase genes were found around the gene cluster in *Paenibacillus* sp. D14 and integrase gene was also found in the downstream of cluster in *Clostridium* sp. D5 (data not shown). Both of them were associated with horizontal gene transfer^35^,^36^. Combining these evidences, *aga1* like gene and surrounding genes in human symbiotic bacteria may have been horizontally transfered from marine bacteria. According to previous work of Hehemann *et al.*^33^, the most possible reason for gene transfer from marine to human microbiota may be seafood diet.

**Figure 5:**
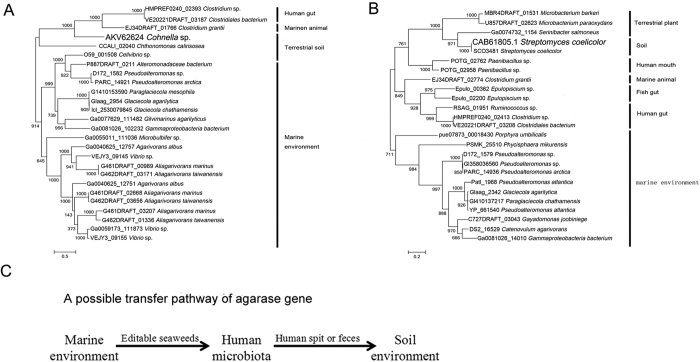
(**A,B**) Maximum likelihood tree of two soil agarases. Numbers at nodes are levels of bootstrap support calculated from 1000 bootstrap replicates. (**C**) The sketch map of the predicted gene transfer pathway.

To further confirm this inferred pathway, phylogenetic trees of two other soil agarases were constructed. As shown in Fig. 5A,B, the same trend could be observed. Soil agarase showed a closer relationship to the agarases of symbiotic environments, such as the human gut or human mouth, than the agarases from marine environments. This evidence also indicated the same mode of gene dissemination; i.e., from marine to symbiotic environments to soil.

Based on these results, we developed the hypothesis that soil agarases may be the result of horizontal gene transfer from a marine environment to a soil environment via human microbiota, and human symbiotic microbiota and human faeces and saliva serving as the link between human microbiota and the soil environment (Fig. 5C).

### Inland human microbiota may use agar

Horizontal gene transfer linkage of marine-symbiont-soil was inferred. Agarases are abundant in marine environments and are found in inland soil environments. However, agarases have not been found in other inland populations, the missing link in the above chain.

**Figure 6:**
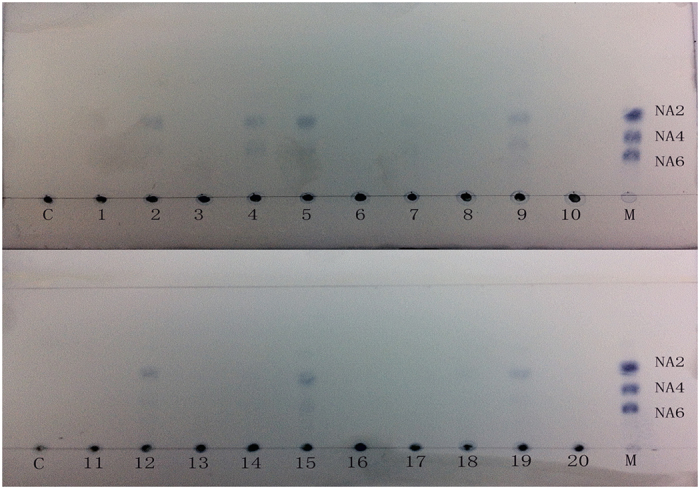
The TLC analysis of the hydrolysis product by inland human fecal sample. *NA2, NA4, NA6*: neoagarobiose, neoagarotetraose, neoagarohexaose.

Thus, we used 37 characterized agarases as queries to investigate their distribution in human symbiotic microbes. Several possible agarases could be found in the microbes of inland people (Table 2). Twenty faecal samples from inland people were used to test for the capacity to degrade agarose. Interestingly, 8 of 20 samples were found to degrade agarose partially (Fig. 6). The screening of agar-degrading bacteria on an agar plate did not achieve positive results, possibly because of unsuitable cultural conditions. Given these results, the microbiota of inland people may also utilize agar.

## Discussion

Until now, most studied agarases have come from marine environments, and very few studies have focused on agarases from terrestrial environments, such as soil. In our previous study, an agarase was purified from the soil agar-degrading bacterium *Paenibacillus* sp. SSG-1^27^, whose genome was subsequently sequenced (data not published). Using the identified peptides (tandem mass result), we found that natural agarase matched a hypothetical protein in the genome. This hypothetical protein showed specific activity against agarose and was thus designated as Aga1. Aga1 showed very low similarity (lower than 30%) to characterized proteins and was thought to be a distant member of GH86.

Aga1 is an exo-type β-agarase, which hydrolyses agarose into NA 4 and NA 6 as end products. In contrast, most exo-agarases produce only one type of oligosaccharide instead of a mixture; for example, Aga50D^37^, Aga21^38^, and AgWH50A^15^ produce NA2 as an end product, and AgWH50C^14^ produces NA4 as an end product. The structure of agarase greatly affects its end products^39^. Thus, the catalytic pattern of Aga1 may be different from those of known agarases.

Aga1 has S-layer homology (SLH) domains that are associated with anchoring to the cell wall surface, and the subcellular prediction results showed that Aga1 was located on the cell wall. Previously, most agarases were intracellular or extracellular^40^. The SLH domain was not found in other reported agarases, nor was a domain with similar function. The cell-wall-binding enzymes were thought to be an efficient way to decompose polysaccharides into smaller components that were suitable for cells to absorb^41^. This was also found in the starch utilization system, which showed multiple functional proteins displayed on the surfaces of bacteria^42^. Thus, Aga1 was possibly located on the cell surface to degrade the agar, which has seldom been reported in agarases.

Given the scarcity of agar in inland environments and the distance between these environments and geographic locations with a sea environment, *aga1* is unlikely to be the result of self-evolution or direct gene transfer from the sea. With the appearance of several agarases in inland symbiotic microbes, inland human microbiota are the most likely source. It has been reported that using human faeces as manure may cause antibiotic resistance gene transfer^43^. Additionally, saliva has also been shown to spread microbes in the environment. The “contamination” of soil with human faeces or saliva maybe the precondition for the gene transfer of agarase in an inland soil environment.

An anomalous nucleotide composition indicated that *aga1* was the result of horizontal gene transfer. In *Paenibacillus* sp. SSG-1, *aga1* and other agar utilization genes, which encoding for NABH, galactosidase and L-AnG metabolic enzymes, were clustered. Meanwhile, gene clusters from different species were compared. The scarcity of agar utilization genes in their corresponding genus, the closest homologues between different microbes, the conserved gene pairs between different taxa and the appearance of transposase and integrase indicated *aga1* may have been transfered together with other genes from marine environments to human microbiota, and to soil environments. This gene cluster transfer was also found in other human gut bacterium^32^. This gene cluster transfer is reasonable, because gene cluster transfer may enable microbes to utilize agar, while transfer of one agarase is not sufficient.

All in all, soil agarase *aga1* may be a result of horizontal gene transfer, from marine environment to soil environment via human microbiota. This gene transfer was also observed with two other soil agarases. In particular, Sco3481, the soil neoagarobiose hydrolase, was founded in microbes from terrestrial plants. This was consistent with the appearance of agar-degrading bacteria in plant associated environments^29^. Additionally, this evidence confirmed that human faeces and saliva affect not only soil but also plants in soil environments. Acquiring agarase may enable soil or plant associated microbes to use agar or agar-like polysaccharides. However, it is still unknown if this gene transfer provides an advantage.

In this study, *aga1* was from an inland bacterium. It is known that microbiota in coastal humans may acquire agarase genes through seafood, which has not been reported in inland populations. If this gene transfer linkage exists, it is reasonable to infer that inland human microbiota have agarase genes.

Using the 37 known agarases as queries, several possible agarases were found in human reference genomes, and some of them were from inland human microbes. In addition, a previous study indicated that agarases from GH86 and GH117 specifically appeared in the human digestive system. Moreover, these agarases were distributed in a North American population, as well as a Japanese population^44^. The lack of studies on agarases has made bioinformatic analysis difficult. As many agarases or seaweed associated genes may be annotated as hypothetical proteins or simple glycoside hydrolases, bioinformatic analysis are restricted. These data suggest that agarases may be distributed in human microbiota, including microbiota from inland people.

In our study, 8 of 20 faecal samples from inland people were shown to have agar-degrading capacity. Given the difficulty in screening for agar-degrading bacteria due to unfavourable culture conditions, these findings are still encouraging. Additionally, a previous study showed that inland people’s microbiota could degrade agar-oligosaccharides and that an agar-degrading bacterium, *B. uniformis* L8, was isolated from inland human faecal samples^31^. These results suggest that microbiota from an inland population can degrade agar.

A previous study of Hehemann *et al.*^33^ showed that seaweed associated genes were horizontally transfered into *Bacteroides plebeius* of the Japanese population, which traditionally eats non-cooked seaweed. Seaweed food, known as “sea vegetables”, is a popular food, not only in coastal regions but also in inland areas^2^,^3^,^4^,^45^. Diet changes have always altered human microbiota^46^, and microbes living inside the body have employed gene transfer to gain functions to adapt to changes^44^. Taking seaweed as food may explain how microbiota from inland people have acquired the agarases from marine bacteria. Meanwhile, agar-degrading bacteria have been reported to produce agar-oligosaccharides with biological functions, which may influence human microbiota^47^. Thus, using seafood as food may influence the balance of human microbiota, which has been shown to be important for human health. Further study is needed to determine whether eating seaweed can affect human microbiota.

## Conclusion

In conclusion, we first cloned and characterized an exo-type β-agarase; i.e., Aga1, from *Paenibacillus* sp. SSG-1. *aga1* showed low similarity to known glycoside hydrolases and may be a distant member of the GH 86 family. *aga1* gene may be the result of horizontal gene transfer from marine environments to humans to soil. Using seaweed as food and human faeces or saliva are the most likely linkages for this gene transfer pathway. Our results indicate that inland human microbiota also have the opportunity to acquire seaweed-associated genes from microbes that attach to the surface of seaweed foods.

## Methods

### Bacterial strains and culture medium

*E. coli* DH5α was used as the general gene-cloning host, and *E. coli* BL21 (DE3) was used as the host for protein expression. Unless otherwise noted, *E. coli* trains were cultured in Luria-Bertani (LB) medium with 100 μg/mL kanamycin. *Paenibacillus* sp. SSG-1 was cultured at 37 °C in LB medium. Strain SSG-1 had been deposited in the China Center Type Culture Collection (CCTCC) with the accession number CCTCC CB 2015001.

### Gene cloning

After overnight culturing, the cell pellet of *Paenibacillus* sp.SSG-1 was harvested, and the genomic DNA was extracted. The *aga1* gene was amplified using high-fidelity PrimeSTAR Max DNA Polymerase (Takara, Japan). The PCR product was digested with *Xhol*I*/Not* I and then ligated into the pET28a vector, which was also digested with *Xhol* I */Not* I. After transformation into *E. coli* BL21, the recombinant plasmid was sequenced to confirm the accuracy of PCR. The sequences of the primers are listed in Supplemental Table S5.

### Domain analysis of Aga1

Conserved domains of Aga1 were analysed using InterPro, and secondary structure analysis was conducted at the PSIPRED site (http://bioinf.cs.ucl.ac.uk/psipred/). Cell-PLos 2.0 was used to predict the subcellular location of Aga1, and signal peptides were predicted using SignalP 3.0.

### Protein production and purification

Recombinant Aga1 was produced with an auto-induction method. *E. coli* BL21 harbouring the recombinant plasmid was cultured in LB medium containing 100 μg/mL kanamycin. After overnight culturing, the *E. coli* cells were inoculated into 2 L auto-induction-medium and then cultured at 28 °C for 48 h. After centrifugation, the cell pellet was collected and then suspended in 50 mL of 20 mM PB buffer with sonication. The supernatant was harvested. The protein was further purified using Ni-column (0.7 × 2.5 cm; GE Healthcare). The elution fractions with agarase activity were collected and further analysed by SDS-PAGE. Unless otherwise noted, the protein purification procedure was conducted at 4 °C.

### Biochemical characterization of Aga1

To analyse the substrate specificity of Aga1, CMC-Na, pectin, carrageenan (mixture of κ, λ and ι), sodium alginate, arabic gum, neoagarooctaose, neoagarotetraose, neoagarohexaose and neoagarooctaose were tested.

To confirm whether Aga1 was correctly produced and purified, purified protein was obtained to conduct tandem mass spectrometry (MS) analysis. A local database was created using the protein data of *Paenibacillus* sp. SSG-1 and the Mascot search engine were used to identify the matched protein. The DNS method was used to assay agarase activity, with D-galactose as the standard. The assay procedure was conducted as previously described. Enzyme activity (U) was defined as the amount of enzyme that liberated 1 μmol D-galactose per minute. The optimal pH and temperature and the stability at different pH values and temperatures were tested as previously described. Various metal ions and chemical reagents (1 mM) were added to the reaction solution to investigate their effects on agarase activity. All experiments were conducted in triplicate.

### Analysis of the degrading pattern of Aga1

To investigate the hydrolysis pattern of Aga1, 100 μg of purified enzyme was added to 50 mL of 0.5% substrate solution (0.5% agarose in deionized water). The reaction solution was incubated at 40 °C. Different samples were collected at fixed intervals. The collected samples were applied to silica G plates (Qingdao Haiyang Chemical Co., Ltd) using n-butyl alcohol:water:acetic acid = 2:1:1 as the developing solvent and then visualized using phenylamine/diphenylamine solution. High performance liquid chromatography (HPLC) was also used to detect the reaction (column HPX87-H Biorad 300 × 7.8 mm).

### Bioinformatic analysis of Aga1 and related agarases

*aga1* similar sequences were obtained from NCBI, Integrated Microbial Genomes (IMG) and the NIH Human Microbiome Project (HMP). Sequences were aligned with the Clustal W program and modified using Gblocks. The phylogenetic tree was constructed using a maximum likelihood method in PhyML. The nucleotide composition and codon usage analyses were conducted using the CodonW online service (http://mobyle.pasteur.fr/cgi-bin/portal.py#forms::CodonW). The species tree was constructed in MEGA 6.0 using a neighbour-joining method. Analysis of other two soil agarases was conducted using the same procedure.

### Agar degrading experiments using microbiomes from an inland population

This study was approved by the Ethics Committee of Sichuan University. 20 persons, who lived in Chengdu, were enrolled to collect stool specimens. Informed consent was obtained from all participants. All experimental procedures were carried out in accordance with the Committee’s approved guidelines. The faecal samples were diluted (0.1 g samples were added to 10 mL of deionized water) and 50 μl of the diluted sample was added into 3 mL of the medium that contained agarose as a sole carbon source. The medium contained 0.1% NaCl, 0.1%K_2_HPO_4_, 0.1% (NH_4_)_2_SO_4_, 0.05% MgSO_4_, 0.01% CaCl_2_, 0.2% yeast extract and 0.2% agarose. After cultivation at 37 °C for 5 days, 50 μl of supernatant was collected and subjected to TLC analysis.

### Investigation of the distribution of agarase in human symbiotic microbes

The protein sequences of 37 characterized agarases were obtained from the CAZy database and were used as search queries. IMG online Blast service (https://img.jgi.doe.gov/cgi-bin/mer/main.cgi?section=FindGenesBlast&page=geneSearchBlast) was used to search for proteins with high similarity (over 40%). Similar proteins from human-related microbes were chosen,and information on geographic locations was also collected.

### Identification of agar utilization proteins

The agar’s utilization enzymes were studied in recent years, proteins in the database may be not annotated. Thus, experimental confirmed enzymes were used as the search queries and proteins with confident identity (higher than 30%) were deduced to have same function. The accession numbers of the search sequences were listed in the Supplemental Table S6.

### Sequence accession number

The nucleotide and protein sequences of the *aga1* gene were submitted to the DDBJ under the accession numbers LC094956 and BAT46645.1, respectively. The accession numbers of *aga1*’s surrounding genes were listed in Supplemental Table S7.

## Additional Information

**How to cite this article**: Song, T. *et al.* Horizontal Transfer of a Novel Soil Agarase Gene from Marine Bacteria to Soil Bacteria via Human Microbiota. *Sci. Rep.* **6**, 34103; doi: 10.1038/srep34103 (2016).

## Supplementary Material

Supplementary Information

## Figures and Tables

**Table 1:** Correspondence analysis of codon usage of *aga1*, *Paenibacillus* sp. SSG-1 and control genes.

	CAI	CBI	Fop	Nc	GC	GC3s
Genome	0.267	0.127	0.489	47.43	0.536	0.633
*aga1*	0.251	-0.029	0.414	52.18	0.457	0.445
PA2609	0.235	0.092	0.468	49.23	0.561	0.634
PA1054	0.217	0.083	0.464	52.60	0.575	0.632
PA4965	0.246	0.013	0.425	57.25	0.511	0.630
PA4486	0.265	0.188	0.508	43.03	0.507	0.631

Control genes were protein-coding genes in *Paenibacillus* sp. SSG-1 and had same GC3s value with the genome. CAI: Codon Adaptation Index. CBI: codon bias index. Nc: effective number of codons. Fop: frequency of optimal codons. GC3s: GC of silent 3rd codon posit.GC: GC content of gene.

**Table 2:** Distribution of the potential agarase genes in human microbiome.

Protein ID	Strain	Identity	Source
BACPLE_01670	*Bacteroide splebeius* DSM 17135	135/286 (47%)	NS
BACPLE_01689	*Bacteroides plebeius* DSM_17135	321/321 (100%)	NS
HMPREF0240_02413	*Clostridium* sp. D5	212/343 (61%)	Inland
HMPREF0240_02442	*Clostridium* sp. D5	155/348 (44%)	Inland
VE20221DRAFT_03240	*Clostridiales bacterium*VE202-21	150/345 (43%)	NS
VE20221DRAFT_03208	*Clostridiales bacterium* VE202-21	198/338 (58%)	NS
VE20221DRAFT_03199	*Clostridiales bacterium* VE202-21	183/321 (57%)	NS
RSAG_01951	*Ruminococcus* sp. 5_1_39BFAA	205/348 (58%)	NS
RSAG_01951	*Ruminococcus* sp. 5_1_39BFAA	205/348 (58%)	NS
RSAG_01951	*Ruminococcus* sp. 5_1_39BFAA	194/347 (55%)	NS
POTG_02958	*Paenibacillus* sp. oral taxon 786	220/352 (62%)	Inland
POTG_02762	*Paenibacillus* sp. oral taxon 786	211/355 (59%)	Inland

Protein IDs are from IMG database. NS: source is not specified.
