# Supplementary material for: Horizontal Transfer of a Novel Soil Agarase Gene from Marine Bacteria to Soil Bacteria via Human Microbiota
Source: Sci Rep. 2016 Oct 19;6:34103. doi: 10.1038/srep34103 (PMC5069468; doi:10.1038/srep34103)
Supplement: Supplementary Information [file srep34103-s1.pdf]

# Horizontal Transfer of a Novel Soil Agarase Gene from Marine Bacteria to Soil Bacteria via Human Microbiota

Tao Song[a*], Hui Xu[a*], Congchong Wei[a], Tengfei Jiang[a], Shishang Qin[a], Weijia Zhang[a], Yu Cao[a,b], Chao Hu[a], Fan Zhang[a], Dairong Qiao[a**], Yi Cao[a**]

Microbiology and Metabolic Engineering of Key Laboratory of Sichuan Province, College of Life Science, Sichuan University, Chengdu, 610065,, P. R. China[a] ; National Engineering Research Center for Biomaterials, Sichuan University, Chengdu, 610065, P. R. China[b].

* Co-first author **Co-Corresponding author;

#Address correspondence to Yi Cao: geneium@scu.edu.cn; Dairong Qiao: qiaodairong@scu.edu.cn;

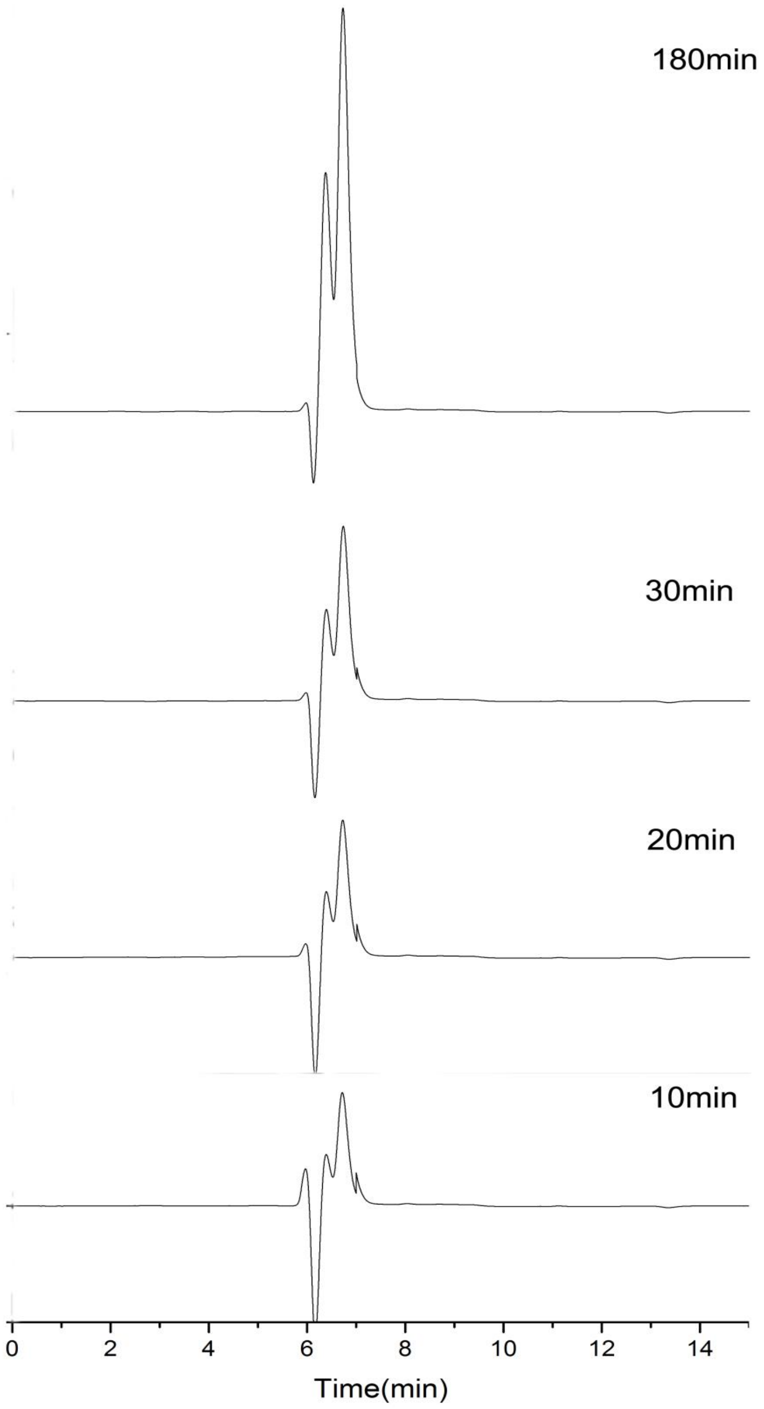


Supplemental Figure S1: HPLC analysis of the hydrolysis product at different time.

Supplemental Table S1: Mascot search result using tandem mass data as query. If Pep_scores of matched peptides were more than 50, the results were confident.

| Matched protein | Pep_score | Sequences of matched peptides |
|---|---|---|
| Aga 1 | 130.61 | AEVVAQDALATDSEINTALEDLTK |
| Aga 1 | 163.32 | YHVNSGPIGVEQADGEITVLDEAYHK |
| Aga 1 | 155.79 | FYVFSPTEEPITNAALAFDR |
| Aga 1 | 56.37 | ISFEDGEAVDYTYNVDTVTTTTDSQGATDGSK |
| Aga 1 | 74.51 | MGFQESNPTEGITHPWLIKDENGDVIYDGYK |
| Aga 1 | 92.28 | WMWENPNSGQSEHFGTPSYSNFDAAADAAAQLIK |

Supplemental Table S2: Effects of various reagents on the activity of Aga 1. Data are mean ± SD of three independent experiments.

| Reagents(1mM) | Relativity activity（%±S.D.） |
|---|---|
| $Na^{+}$ | 107.57±4.14 |
| $K^{+}$ | 104.93±2.13 |
| $Mn^{2+}$ | 93.54±16.89 |
| $Cu^{2+}$ | 37.87±1.94 |
| $Zn^{2+}$ | 83.51±5.71 |
| $Ba^{2+}$ | 113.57±8.62 |
| $Ca^{2+}$ | 99.24±9.70 |
| $Fe^{3+}$ | 104.31±3.10 |
| $Ni^{+}$ | 102.86±1.43 |
| $Mg^{2+}$ | 111.51±2.33 |
| $Pb^{2+}$ | 96.58±6.93 |
| $Co^{2+}$ | 95.69±3.80 |
| β-Me | 122.33±7.18 |
| DTT | 133.44±11.59 |
| SDS | 118.84±10.08 |
| EDTA | 104.02±7.02 |
| Urea | 97.74±11.24 |
| Control | 100.00±12.95 |

Supplemental Table S3: GC content variety and GC3 content variety between Aga 1 and corresponding genome

| ID | GC content variety | GC3s content variety |
|---|---|---|
| Aga 1 | 8.72 | 19.60 |

Supplemental Table S4: Analysis of the RSCU difference between Aga 1 and the

genome, RSCU represents relative synonymous codon usage

| | Code | Aga 1 RSCU | Genome RSCU | | Code | Aga 1 RSCU | Genome RSCU |
|---|---|---|---|---|---|---|---|
| Ala | GCA | 1.3037 | 0.606 | Cys | UGC | 0 | 1.538 |
| | GCC | 0.7111 | 1.4822 | | UGU | 2 | 0.462 |
| | GCG | 1.1556 | 1.3331 | Asp | GAC | 0.6281 | 1.02 |
| | GCU | 0.8296 | 0.5787 | | GAU | 1.3719 | 0.981 |
| Gly | GGA | 1.1171 | 0.8914 | Glu | GAA | 1.0323 | 1.003 |
| | GGC | 1.5856 | 1.7346 | | GAG | 0.9677 | 0.997 |
| | GGG | 0.3243 | 0.8198 | Phe | UUC | 1.2239 | 1.016 |
| | GGU | 0.973 | 0.5541 | | UUU | 0.7761 | 0.984 |
| Leu | CUA | 0.3652 | 0.1564 | His | CAC | 0.8148 | 0.739 |
| | CUC | 0.3652 | 0.9367 | | CAU | 1.1852 | 1.261 |
| | CUG | 1.6696 | 2.9811 | Ile | AUA | 0.4369 | 0.198 |
| | CUU | 1.4609 | 0.7406 | | AUC | 0.7282 | 1.721 |
| | UUA | 0.8348 | 0.3268 | | AUU | 1.835 | 1.082 |
| | UUG | 1.3043 | 0.8585 | Met | AUG | 1 | 1 |
| Lys | AAA | 0.8837 | 0.9163 | Pro | CCA | 1.3731 | 0.369 |
| | AAG | 1.1163 | 1.0837 | | CCC | 0.1791 | 0.677 |
| Asn | AAC | 0.6733 | 1.2099 | | CCG | 1.1343 | 2.318 |
| | AAU | 1.3267 | 0.7901 | | CCU | 1.3134 | 0.636 |
| Arg | AGA | 2.2642 | 0.5212 | Ser | AGC | 1.6034 | 1.805 |
| | AGG | 0.1132 | 0.5075 | | AGU | 0.931 | 0.385 |
| | CGA | 0.6792 | 0.3655 | | UCA | 0.6724 | 0.393 |
| | CGC | 1.2453 | 1.78 | | UCC | 1.2414 | 1.587 |
| | CGG | 0.4528 | 2.0344 | | UCG | 0.9828 | 1.385 |
| | CGU | 1.2453 | 0.7913 | | UCU | 0.569 | 0.445 |
| SeCys | ACA | 1.4444 | 0.5055 | Val | GUA | 0.8254 | 0.569 |
| | ACC | 0.4444 | 1.344 | | GUC | 0.6349 | 1.323 |
| | ACG | 1.3056 | 1.8481 | | GUG | 1.1429 | 1.423 |
| | ACU | 0.8056 | 0.3024 | | GUU | 1.3968 | 0.685 |
| Gly | CAA | 0.7347 | 0.5766 | Tyr | UAC | 0.6111 | 0.988 |
| | CAG | 1.2653 | 1.4234 | | UAU | 1.3889 | 1.012 |
| Trp | UGG | 1 | 1 | Ter | UAG | 3 | 0.589 |

Supplemental Table S5: The primers used for Aga 1. The restrict enzyme sites were red colored.

| Name | Sequence of oligosaccharide primer |
|---|---|
| Aga(F) | ATTTGCGGCCGCGTTGAGGCAAGCGA |
| Aga(R) | CCGCTCGAGAATGACGGCGTAAGTGC |

Supplemental Table S6: Accession number and origin of the query protein

| Gene function | Accession Number | Origin |
|---|---|---|
| α-neoagarobiose hydrolase | WP_013995985.1 | *Zobellia galactanivorans* |
| galactosidase | WP_014232195.1 | *Vibrio sp.* EJY3 |
| 3,6-anhydro-L-galactonate cycloisomerase | WP_014232231.1 | *Vibrio sp.* EJY3 |
| 2,5-diketo-3-deoxy-L-galactonate 5-reductase | WP_011575334.1 | *Pseudoalteromonas atlantica* |
| 3,6-anhydro-L-galactose dehydrogenase | WP_014232205.1 | *Vibrio sp.* EJY3 |
| 2-keto-3-deoxy-L-galactonate 5-dehydrogenase | WP_011575334.1 | *Pseudoalteromonas atlantica* |

Supplemental Table S6: Accession numbers of Aga's surrounding proteins

| Gene locus | Gene function | Accession number |
|---|---|---|
| 7137 | transposase | LC164143 |
| 7138 | transposase | LC164144 |
| 7142 | α-neoagarobiose hydrolase | LC164136 |
| 7144 | 3,6-anhydro-L-galactonate cycloisomerase | LC164138 |
| 7145 | 2,5-diketo-3-deoxy-L-galactonate 5-reductase | LC164141 |
| 7146 | 3,6-anhydro-L-galactose dehydrogenase | LC164140 |
| 7155 | 3,6-anhydro-L-galactose dehydrogenase | LC164137 |
| 7156 | galactosidase | LC164139 |
| 7158 | sulfatase | LC164142 |
